# Secondary traumatic stress among healthcare workers in Egypt and the mediating role of workplace protective factors

**DOI:** 10.1038/s41598-026-61079-4

**Published:** 2026-07-17

**Authors:** Asmaa Ali El-Feky, Mohammed Salama Abuzeid, Asmaa Abdel-Reheem Atalla, Nouran Mohammed El Korashy, Rabab Ahmed Hammad, Amira Kamar Eldawla Mokhtar El-Shenawy

**Affiliations:** 1 Occupational medicine at Department of Public Health and Community Medicine, Faculty of Medicine, Tanta University, Tanta, Egypt; 2 Department of Public Health and community medicine, Faculty of Medicine, Tanta University, Tanta, Egypt

**Keywords:** Secondary traumatic stress, Healthcare workers, Associated factors, Workplace interventions, Diseases, Health care, Health occupations, Medical research, Risk factors

## Abstract

Healthcare workers are occupationally exposed to traumatic events, placing them at risk of secondary traumatic stress (STS) a condition characterized by intrusion, avoidance, and arousal symptoms. To determine the prevalence of STS among healthcare workers, identify early warning signs, evaluate the effectiveness of workplace preventive measures, and identify factors associated with STS. A cross-sectional study was conducted from October to December 2025 among 783 healthcare workers in critical care units at Tanta University Hospitals, Egypt. Data were collected using a structured self-administered questionnaire including the Secondary Traumatic Stress Scale (STSS), an early warning signs scale, and a preventive measures scale. Multivariate logistic regression was used to identify associated factors with STS. The frequency of STS was 64.5%, with a total mean score of 58.16 ± 11.18. The most frequent early warning signs were chronic exhaustion (3.43 ± 1.126), increased anxiety about safety (3.18 ± 1.156), and difficulty maintaining work-life boundaries (3.17 ± 1.206). A strong positive correlation was observed between STS score and warning signs score (*r* = 0.76, *p* < 0.01). Multivariate logistic regression identified physicians (AOR = 2.14), working > 40 h/week (AOR = 2.07), > 8-night shifts/month (AOR = 2.56), absence of preventive measures (AOR = 2.87), and sick leave history (AOR = 1.89) as significant associated factors of severe STS. Mediation analysis revealed that social support mediated the relationship between occupational stressors and secondary traumatic stress, with a significant indirect effect (0.105, 95% CI: 0.062–0.148) accounting for 23.4% of the total effect. STS is highly prevalent among Egyptian healthcare workers and is significantly associated with modifiable workplace factors. The absence of essential organizational interventions highlights critical gaps in occupational health protection. Routine surveillance, mandatory debriefing, and enhanced organizational support are urgently needed.

##  Introduction

Secondary traumatic stress (STS) is defined as the behavioral and emotional consequences of resulting from a traumatic event that another person has experienced due to indirect exposure to traumatic events^1^. Healthcare personnel are frequently exposed to work-related traumatic events, including patient deaths and critical incidents, often indirectly through caring for traumatized patients. Workers in high-acuity settings such as emergency departments, critical care units, and cardiac care units are particularly at increased risk^2^. STS is becoming recognized as an occupational hazard among the health professions, different from burnout and depression but strongly related to trauma exposure^2,3^.

STS symptoms are like those of post-traumatic stress disorder (PTSD) and can be classified into three categories: intrusion, avoidance, and arousal^4^. STS prevalence has been shown to range between 30% and 80% in various healthcare jobs around the world^1,5^. In Saudi Arabia, both high and severe levels of STS were reported, with 27.6% reporting high and 50.8% reporting severe levels^4^. During the COVID-19 pandemic, Egyptian newborn intensive care unit staff reported significant stress symptoms^6^.

Several key problems have been identified in studies examining STS among critical care health care workers, including emotional exhaustion and burnout, depression and anxiety, reduced job satisfaction, decreased patient care quality, and high turnover rates^7^. Workplace determinants of STS include high workload, prolonged shifts, inadequate staffing, lack of organizational support, and insufficient post-incident debriefing^8^. The consequences extend beyond individual health, affecting job performance, patient safety, and healthcare system stability^9^. Despite this, STS remains under-recognized in occupational health surveillance, particularly in low- and middle-income countries where mental health resources are limited.

In Egypt, no comprehensive occupational health assessment of STS among healthcare personnel has taken place. To our knowledge’s, no prior Egyptian study has extensively assessed the availability and efficacy of workplace preventive measures from the perspective of healthcare professionals, indicating a considerable gap in understanding organizational strengths and weaknesses. The present studies have primarily focused on individual coping techniques rather than modifiable workplace variables, thus diverting attention away from institutional commitments to promote worker satisfaction.

This study discusses these gaps by systematically assessing STS frequency across multiple multidisciplinary departments, identifying early warning signs that can be routinely monitored, quantifying the availability and perceived effectiveness of both occupational and personal preventive measures, identifying modifiable workplace factors associated with STS, and investigating the mediating role of social support. This study provides the first comprehensive occupational health assessment of STS among Egyptian healthcare workers, establishing the starting point for benchmarking, informing evidence-based occupational health policy, and positioning STS as a preventable occupational hazard rather than an unavoidable consequence of healthcare work. Therefore, this study aimed to assess the frequency of STS and early warning signs of STS among healthcare workers at Tanta University Hospitals, evaluate the availability and perceived effectiveness of existing personal and occupational preventive measures and identify occupational and demographic factors associated with STS.

## Methods

### Study design, study setting and duration

A cross-sectional observational study was conducted. The study was carried out at critical care units (Emergency Department, Cardiac Care Unit, Surgery, internal medicine, Burns Unit, and Obstetrics and Gynecology) of Tanta University Hospitals, Gharbia Governorate, Egypt, from October to December 2025.

### Study population

The target population included hospital workers, such as nurses and resident physicians. Residents and nurses employed at the chosen hospital were eligible for inclusion, whereas other types of healthcare personnel were excluded. During the study period, about 1180 medical residents registered in Tanta University Hospitals’ 5-year residency program. Nursing staff members worked in several departments in rotating shifts, including 1090 nurses.

### Sample size and sampling strategy

#### Sample size calculation

The sample size was calculated using Epi-Info version 7.2 (Centers for Disease Control and Prevention, Atlanta, GA, USA). The target group included all healthcare personnel (1,180 physicians and 1,090 nurses; total *N* = 2,270) working in Tanta University Hospitals’ critical care units. The sample size was obtained using the following parameters: a predicted STS prevalence of 65% based on a recent meta-analysis by XU et al., (2024) about the Prevalence and associated factors of secondary traumatic stress in emergency nurses^10^, a 95% confidence level, a 5% margin of error, and a design effect of 2 to account for occupation clustering. These criteria resulted in a minimum sample size of 699 people. To account for expected non-response and incomplete questionnaires, the target was adjusted by 12%, yielding a final target sample of 783 individuals.

#### Sampling strategy

A two-stage stratified sampling strategy was used. To guarantee proportional representation, participants were first stratified by profession (physician, nurse). In the second step, each professional stratum was further divided by clinical department (emergency department, burn unit, surgery, internal medicine, cardiology, and obstetrics and gynecology), to ensure adequate representation where STS risk is known to be high. Participants were recruited from each stratum using a convenience sampling method until the proportional allocation target was fulfilled.

**Fig. 1:**
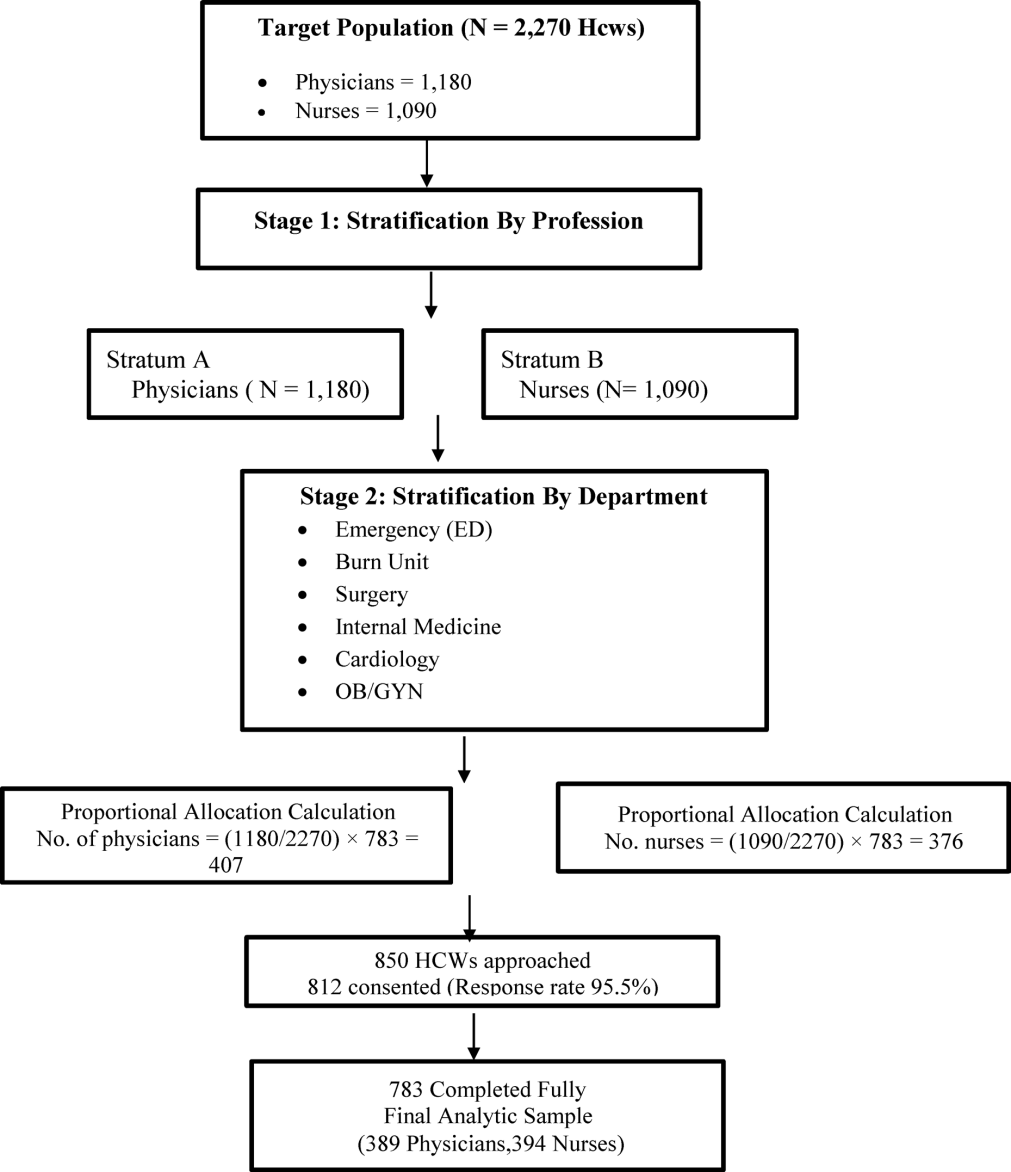
Flowchart demonstrating the sampling strategy.

A total of 850 healthcare workers were approached during the study period. Of these, 812 provided consent (response rate 95.5%), and 783 completed the questionnaire in full, yielding a final analytic sample of 783 participants. The sample comprised 389 physicians (49.7%) and 394 nurses (50.3%), consistent with the proportional distribution of these professions in the target population (Fig. 1).

### Data collection tools

Data was collected using a self-administered structured questionnaire, consisting of:

Demographic and occupational data: age, sex, profession, years of experience, night shifts per month, working hours per week, department, history of sick leave due to psychological trauma, prior mental health consultation, and perceived social and organizational support.

Secondary traumatic stress scale (STSS): the STS scale is a 17-item survey with a five-point Likert scale ranging from 1 to 5, used to assess the frequency of STS symptoms produced by indirect exposure to traumatic events experienced by others. The questionnaire was designed to assess the intrusion, avoidance, and arousal symptoms associated with indirect exposure to traumatic events^11^. In the original validation study, the STSS demonstrated good internal consistency (total α = 0.93; Intrusion α = 0.80; Avoidance α = 0.87; Arousal α = 0.83), as well as convergent, discriminant, and factorial validity^11^. The tool was translated into Arabic and the validity and reliability, or Arabic version was calculated (Cronbach’s α = 0.87).

A total STSS score was computed as the sum of the 17 item scores (possible range: 17–85). The established cutoff of > 37, derived from previous validation studies^12,13^, was used to categorize participants as meeting the criteria for STS. This cutoff has been widely employed in international occupational health research^14^ and demonstrated strong internal consistency in our sample (Cronbach’s α = 0.94), supporting its suitability for this Egyptian healthcare population.

Early warning signs scale: an 11-item checklist assessing frequency of STS-related symptoms on a 5-point Likert scale. This tool was developed by researchers by reviewing previous literature^13,14^. The total score ranges from 11 to 55. The tool was translated to Arabic using translation and back translation. To ensure tool validity, it was sent to four experts for face and content validity. The content validity index was calculated and reported as 85.5%.

Preventive measures scale: the preventive measures scale was developed by researchers to evaluate the availability and perceived effectiveness of strategies aimed at mitigating STS among healthcare workers. The scale was adapted and modified from Al Barmawi et al.^13^, with additional items derived from the occupational health literature on trauma-exposed professionals^8,9^.

The items were chosen using two conceptual frameworks: (1) the occupational health hierarchy of controls, which emphasizes organizational-level interventions over individual-level coping strategies, and (2) the Job Demands-Resources (JD-R) model, which proposes that workplace resources ( social support, development opportunities) buffer the negative effects of job demands on psychological well-being^15^. Items were categorized into two domains:

- Occupational preventive measures (7 items): organizational and workplace-level interventions including debriefing sessions, financial incentives, break time post-exposure, development opportunities/training, assignment shift rotation, support from colleagues, and psychological support services.
- Personal preventive measures (4 items): individual-level coping strategies including prayer and spiritual activities, spending time with others/family, adequate sleep, and recreation activities.

A panel of four experts from occupational health, mental health nursing, and public health evaluated the content validity. The item-level Content Validity Index (I-CVI) varied between 0.75 and 1.00, whereas the scale-level Content Validity Index (S-CVI/Ave) was 0.85, indicating appropriate content. Cronbach’s alpha coefficient was used to assess internal consistency reliability for both the whole scale and each domain separately. The overall scale had strong internal consistency (α = 0.82). The occupational domain demonstrated acceptable internal consistency (α = 0.79), in accordance with earlier research^13^. The personal domain showed acceptable reliability (α = 0.71). Participants were asked to score each preventive measure based on their personal experiences and perceptions from the previous 12 months. A total effective sum score was generated by adding scores for all 11 questions (ranging from 0 to 33), with higher scores indicating better perceived effectiveness of available preventive strategies^13,16^.

### Statistical analysis

The data was analyzed with SPSS Version 27. Test on normality was done by Shapiro-Wilk test and the data was normally distributed. For continuous variables, descriptive statistics were calculated as means ± standard deviations, whereas categorical variables were represented by frequencies and percentages. Bivariate analysis was carried out using chi-square tests for categorical variables and independent t-tests for continuous variables. A multivariate logistic regression analysis was performed to discover characteristics that are independently related to STS (STSS score > 37). The adjusted odds ratios (AOR) were estimated using 95% confidence intervals. Statistical significance was determined at *p* < 0.05. To investigate the mediating role of social support in the association between occupational stressors and STS, the PROCESS Macro for SPSS (Model 4) was used. Occupational stressors have been implemented as a composite score calculated by adding the standardized ratings for weekly working hours and monthly night shifts. The indirect effect was investigated using bootstrapping with 5,000 resamples to create 95% bias-corrected confidence intervals. An indirect effect was judged statistically significant if the confidence interval did not include zero.

## Results

### Participant characteristics

The study sample of 783 healthcare workers was mostly female (58.5%) and had an average age of around 30 years. The participants were almost evenly distributed between physicians (49.7%) and nurses (50.3%), with the majority having a bachelor’s degree as their highest academic level (58.4%). In terms of clinical settings, most participants worked in emergency departments (23.0%), followed by burn units (17.5%) and surgery (16.1%). The staff was very experienced, with an average of 6.37 years of practice, but they also reported difficult work schedules, which included 47.4 working hours per week and 9.3-night shifts per month (Table 1).

**Table 1:** Socio-demographic and occupational characteristics of study participants (*N* = 783).

Characteristic	Category	Frequency (*n*)	Percentage (%)
Age (years)	Mean ± SD	29.86 ± 6.64	
Sex	Male	325	41.5
	Female	458	58.5
Residence	Urban	358	45.7
	Rural	425	54.3
Marital status	Single	361	46.1
	Married	407	52.0
	Divorced / widowed	15	1.9
Profession	Physician	389	49.7
	Nurse	394	50.3
Highest academic degree	Diploma	134	17.1
	Bachelor	457	58.4
	Master	192	24.5
Department	Emergency	180	22.99
	Burn Unit	137	17.50
	Surgery	126	16.09
	Internal Medicine	112	14.30
	Cardiology	87	11.11
	Other (burn & obstetrics and gynecology)	75	9.58
Years of experience	Mean ± SD	6.37 ± 7.27	
Working hours/week	Mean ± SD	47.39 ± 21.50	
Night shifts/month	Mean ± SD	9.32 ± 4.28	

### Secondary traumatic stress scale and warning signs among health care workers

Table 2 shows that the total mean score of secondary traumatic stress among healthcare workers was 58.16 ± 11.18. The avoidance subscale had the greatest mean (22.57 ± 7.98). This is followed by arousal (19.05 ± 5.31), with elevated ratings in items such as feeling jumpy (3.91 ± 1.10), easily annoyed (3.85 ± 1.01), and difficulty sleeping (3.83 ± 1.02). Intrusion had a mean of 16.54 ± 4.04, with frequent unintentional thoughts about work (3.69 ± 1.07), reliving clients’ trauma (3.54 ± 1.09), and disturbing dreams (3.55 ± 1.20). The average warning sign score was 30.10 ± 7.562. The most notable warning indicators included chronic exhaustion (3.43 ± 1.126), increased anxiety or concern about safety (3.18 ± 1.156), and difficulties maintaining work-life boundaries (3.17 ± 1.206). Lower ratings were found for anger or cynicism towards work (2.17 ± 1.100) and perceived trauma from patient care (2.45 ± 1.124).

**Table 2:** Secondary traumatic stress scale and warning signs among health care workers.

Subscale / item	Mean	SD
**Intrusion**	16.54	4.04
1. Experienced heart pounding when thinking about working with clients	2.38	0.99
2. Getting upset when being reminded of work with clients	3.38	0.89
3. Feeling like reliving the trauma experienced by the clients	3.54	1.09
4. Thinking about the work with clients when not intending	3.69	1.07
5. Having disturbing dreams about work with clients	3.55	1.20
**Arousal**	19.05	5.31
6. Trouble sleeping	3.83	1.02
7. Feeling jumpy	3.91	1.10
8. Easily annoyed	3.85	1.01
9. Trouble concentrating	3.69	1.09
10. Expecting something bad to happen	3.77	1.09
**Avoidance**	22.57	7.98
11. Feeling emotionally numb	3.70	1.18
12. Feeling discouraged about the future	3.07	1.23
13. Having little interest in being around others	3.70	1.18
14. Avoiding people, places, or things that remind you of working with clients	3.85	1.16
15. Feeling less active than usual	2.67	1.14
16. Wanting to avoid working with some clients	2.73	1.13
17. Noticing gaps in the memory about client sessions	2.85	1.16
Total score	58.16 ± 11.18 (range: 17–85)	
**Warning signs**		
1. Increased anxiety or concern about safety	3.18	1.156
2. Regularly feeling angry and/or cynical about staff and your work	2.17	1.100
3. Startle when hearing loud sounds	2.91	1.181
4. Avoiding people, enjoyable places, and activities	2.53	1.077
5. Feeling emotionally numb or disconnected	2.48	1.130
6. Feelings of chronic exhaustion	3.43	1.126
7. Not remembering important details	2.61	1.065
8. Intrusive, negative thoughts and images	2.49	1.157
9. Difficulty maintaining work-life boundaries	3.17	1.206
10. Feeling depressed after caring for patients	2.67	1.158
11. Feeling that I had trauma when caring for trauma patients	2.45	1.124
Total warning signs score	30.10	7.562

**Fig. 2:**
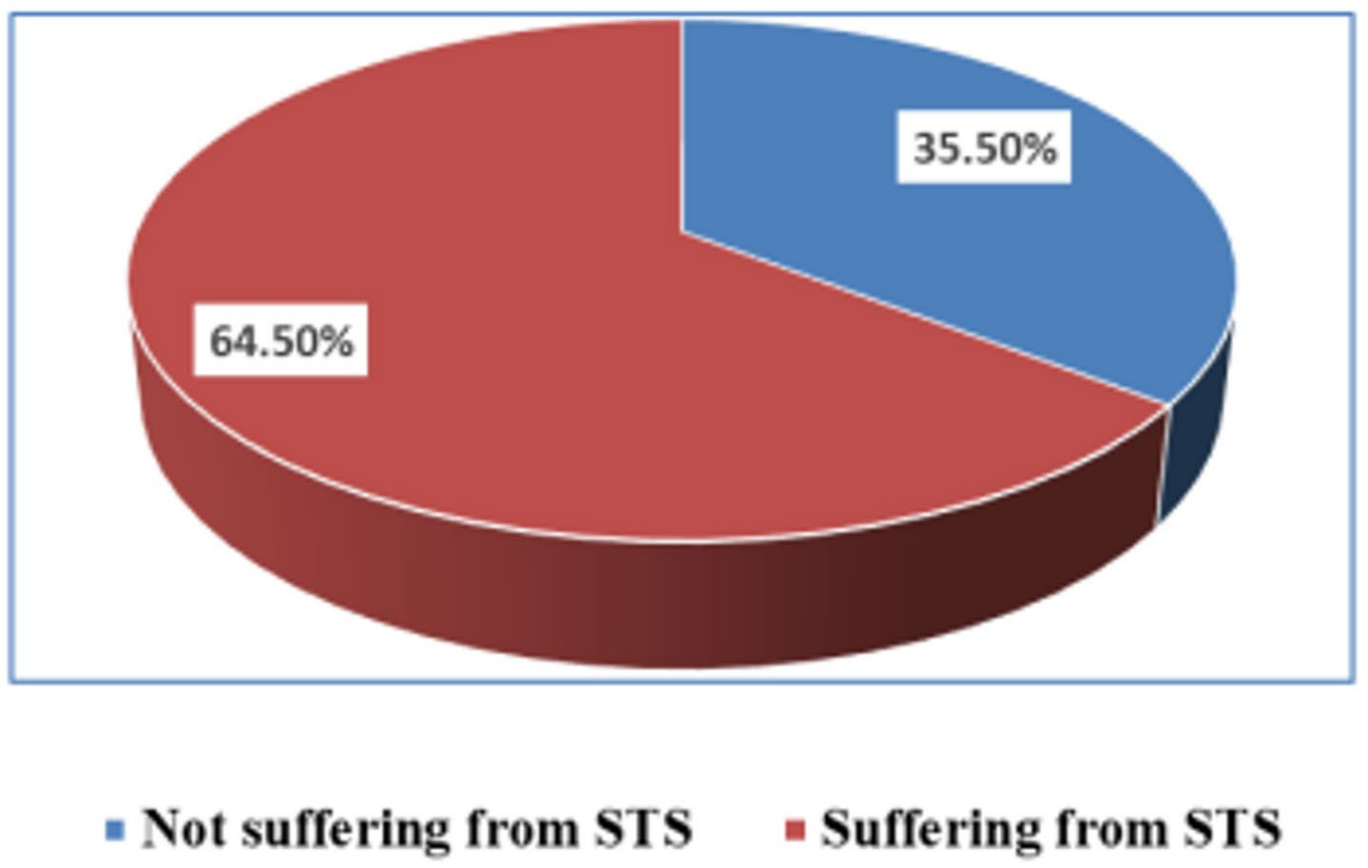
Prevalence of secondary traumatic stress among healthcare workers.

The established cutoff (> 37), 64.5% (*n* = 505) of participants were identified as suffering from STS, while 35.5% (*n* = 278) did not meet the threshold (Fig. 2).

###  Preventive strategies

Table 3 presents the availability and perceived effectiveness of occupational and personal preventive measures for mitigating secondary traumatic stress (STS) among healthcare workers. Among occupational interventions, debriefing sessions were largely absent (71.6%), yielding the lowest mean effectiveness score (0.44 ± 0.789). Support from colleagues was unavailable for 46.5% (mean 0.91 ± 0.994), and development opportunities were absent for 43.4% (mean 0.98 ± 1.023). Psychological support services were predominantly rated as ineffective (55.4%), despite universal availability. Financial incentives showed the highest occupational effectiveness (mean 1.78 ± 0.940), followed by break time post-exposure (1.70 ± 0.899) and shift rotation (1.43 ± 1.014).

Personal preventive measures demonstrated substantially higher perceived effectiveness. Adequate sleep received the highest mean score (2.24 ± 0.726), followed by recreation activities (2.07 ± 0.758). Prayer and spiritual activities were rated very effective by the highest proportion (50.4%, mean 1.95 ± 0.744). The total effectiveness sum score was 16.89 ± 5.32, reflecting reliance on individual coping strategies in the absence of adequate organizational support.

**Table 3:** Availability and effectiveness of occupational preventive measures and personal preventive measures.

Preventive measures	Not present No. (%)	Ineffective No. (%)	Partially effective No. (%)	Very effective No. (%)	Mean *±* SD
**Occupational measure**					
Debriefing sessions	562 (71.6%)	124 (15.8%)	75 (9.6%)	24 (3.1%)	0.44 ± 0.789
Financial incentives	110 (14.0%)	120 (15.3%)	385 (49.0%)	170 (21.7%)	1.78 ± 0.940
Break time post-exposure	98 (12.5%)	174 (22.2%)	376 (47.9%)	137 (17.5%)	1.70 ± 0.899
Development opportunities/training	341 (43.4%)	190 (24.2%)	179 (22.8%)	75 (9.6%)	0.98 ± 1.023
Assignment shift rotation	198(25.2%)	159(20.3%)	319 (40.6%)	109 (13.9%)	1.43 ± 1.014
Support from colleagues	365 (46.5%)	184 (23.4%)	176 (22.4%)	60 (7.6%)	0.91 ± 0.994
Psychological support services	2 (0.3%)	435 (55.4%)	264 (33.6%)	84 (10.7%)	1.55 ± 0.684
**Personal preventive measures**					
Prayer and spiritual activities	2 (0.3%)	126 (16.1%)	261 (33.2%)	396 (50.4%)	1.95 ± 0.744
Spending time with others/family	1 (0.1%)	299 (38.1%)	312 (39.7%)	173 (22.0%)	1.84 ± 0.762
Enough sleep	1 (0.1%)	132 (16.8%)	330 (42.0%)	322 (41.0%)	2.24 ± 0.726
Recreation activities	0 (0.0)	199 (25.4%)	330 (42.0%)	256 (32.6%)	2.07 ± 0.758
Total effectiveness (sum score)					16.89 *±* 5.32

### Associated factors with secondary traumatic stress

Table 4 presented the associations between demographic/occupational factors and secondary traumatic stress (STS) severity. Females reported significantly higher mean STS scores than males (60.44 ± 10.72 versus 49.56 ± 10.23, *p* < 0.001). Among occupational factors, physicians exhibited the highest STS scores (62.83 ± 11.47) compared to nurses (56.44 ± 10.72, *p* < 0.001). Department was also significantly associated (*p* < 0.001), with Burn Unit (65.64 ± 12.89) and Emergency Department (62.18 ± 11.23) personnel reporting the highest levels. Workload-related factors were strongly associated with STS severity; participants working > 40 h per week (63.21 ± 11.87 vs. 56.98 ± 10.84, *p* < 0.001) and those with > 8-night shifts per month (61.89 ± 11.23 vs. 57.32 ± 10.95, *p* < 0.001) reported significantly higher scores. Individuals who had taken sick leave also demonstrated higher STS scores (65.21 ± 11.87 vs. 52.98 ± 10.84, *p* < 0.001). No significant associations were found with residence, marital status, or academic degree.

**Table 4:** Associations between demographic/occupational factors and secondary traumatic stress (STS) severity among the study participants.

Variable	Category	Mean STS score ± SD	*p*-value
Sex	Male	49.56 ± 10.23	< 0.001*
	Female	60.44 ± 10.72	
Residence	Urban	58.96 ± 10.23	0.87
	Rural	58.44 ± 10.72	
Marital status	Married	59.21 ± 10.87	0.08
	Single	49.76 ± 10.20	
	Divorced/widowed	50.98 ± 10.65	
Profession	Physician	62.83 ± 11.47	< 0.001*
	Nurse	56.44 ± 10.72	
Highest academic degree	Diploma	58.89 ± 11.09	0.32
	Bachelor’s	57.32 ± 10.50	
	Postgraduate	59.89 ± 9.23	
Department	Burn unit	65.64 ± 12.89	< 0.001*
	Emergency	62.18 ± 11.23	
	Surgery	50.04 ± 11.56	
	Others	47.87 *±* 9.87	
Working hours/week	> 40 h	63.21 ± 11.87	< 0.001*
	≤ 40 h	56.98 ± 10.84	
Night shifts/month	> 8 shifts	61.89 ± 11.23	< 0.001*
	≤ 8 shifts	57.32 ± 10.95	
Sick leave	Yes	65.21 ± 11.87	< 0.001*
	No	52.98 ± 10.84	

*p < 0.05.

**Fig. 3:**
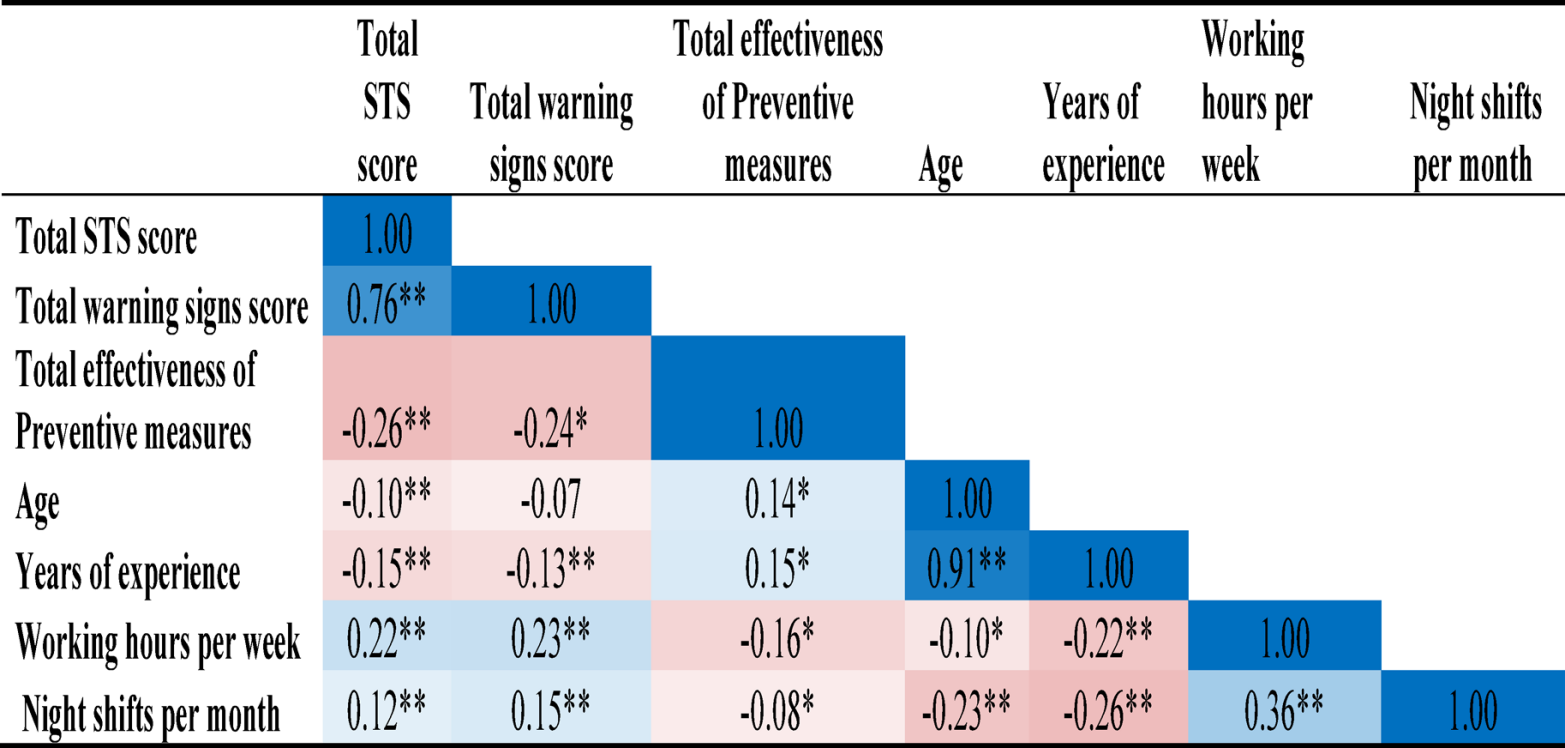
Correlation matrix of study variables. **p < 0.01.

Figure 3 showed that a strong positive correlation was observed between STS score and warning signs score (*r* = 0.76, *p* < 0.01), indicating that higher STS severity was associated with more frequent early warning signs. Total effectiveness of preventive measures showed significant negative correlations with both STS (*r* = -0.26, *p* < 0.01) and warning signs (*r* = -0.24, *p* < 0.05). Age and years of experience demonstrated weak negative correlations with STS (*r* = -0.10 and − 0.15, respectively) and positive correlations with preventive measure effectiveness (*r* = 0.14 and 0.15). Working hours per week and night shifts per month showed positive correlations with STS (*r* = 0.22 and 0.12) and warning signs (*r* = 0.23 and 0.15), and negative correlations with preventive measure effectiveness (*r* = -0.16 and − 0.08).

Table 5 showed the Multivariate logistic regression analysis to identify independent factors associated with secondary traumatic stress (STS) severity. The model demonstrated adequate fit (Hosmer-Lemeshow χ² = 6.24, *p* = 0.621) and explained 23.8% of the variance (Nagelkerke R² = 0.238). Physicians had more than twice the odds of severe STS compared to nurses (AOR = 2.14, *p* = 0.002). Workload factors were strongly associated, working > 40 h per week (AOR = 2.07, *p* < 0.001) and > 8-night shifts per month (AOR = 2.56, *p* < 0.001) significantly increased odds. The absence of occupational preventive measures was the strongest associated factor, conferring nearly three times the odds of severe STS (AOR = 2.87, *p* < 0.001). History of sick leave due to psychological trauma was also associated with nearly double the odds (AOR = 1.89, *p* = 0.005). Age (AOR = 0.99, *p* = 0.524) and sex (AOR = 1.18, *p* = 0.486) were not significant in the multivariate model.

**Table 5:** Multivariate logistic regression analysis of factors associated with STS severity score.

Variable	Category	AOR	95% CI	*p*-value
Profession	Physician (vs. nurse)	2.14	1.32–3.48	0.002
Working hours/week	> 40 (vs. ≤40)	2.07	1.71–4.82	< 0.001
Night shifts/month	> 8 (vs. ≤8)	2.56	1.58–4.15	< 0.001
Occupational preventive measures	Not present (vs. effective)	2.87	1.71–4.82	< 0.001
Sick leave	Yes (vs. no)	1.89	1.21–2.95	0.005
Age	Per 1-year increase	0.99	0.96–1.02	0.524
Sex	Female (vs. male)	1.18	0.74–1.89	0.486

*Model fit: Hosmer-Lemeshow χ² = 6.24, *p* = 0.621; Nagelkerke R² = 0.238.

Table 6 presents the mediating role of support in the relationship between occupational stressors and secondary traumatic stress (STS), along with the various workplace and personal preventive measures. Mediation analysis revealed that social support partially mediated the relationship between occupational stressors and secondary traumatic stress, with a significant indirect effect (0.105, 95% CI: 0.062–0.148) accounting for 23.4% of the total effect. Among occupational preventive measures, financial incentives, assignment shift rotation, and support from colleagues demonstrated significant protective effects, reducing STS scores by 1.16 to 1.23 points (all *p* < 0.01). Conversely, psychological support services showed a paradoxically positive association with STS (coefficient = 1.27, *p* = 0.02). Personal preventive measures including prayer, sleep, and recreation did not reach statistical significance in the multivariate model, underscoring that organizational-level interventions may be more critical than individual coping strategies for mitigating STS among healthcare workers.

**Table 6:** Mediating role of protective effects of workplace and personal factors on STS.

Variable	Coefficient	SE	95% CI	Test statistic	*p*-value
**Mediation model**					
Direct effect (occupational stressors on STS)	0.342	0.051	0.242–0.442	6.71	< 0.001
Indirect effects (stressors and protective effects on STS)	0.105	0.022	0.062–0.148	4.77	< 0.001
Total effect	0.447	0.058	0.333–0.561	7.71	< 0.001
Proportion mediated	23.4%				< 0.001
**Protective effects**					
**Occupational preventive effects**					
Debriefing sessions	0.114	0.52	-0.92–1.14	0.21	0.82
Financial incentives	-1.23	0.43	-2.08 – -0.37	-2.82	0.004*
Break time post-exposure	-0.92	0.48	-1.87–0.03	-1.89	0.058
Development opportunities/training	-0.49	0.45	-1.39–0.39	-1.09	0.27
Assignment shift rotation	-1.20	0.40	-2.00 – -0.40	-2.94	0.003*
Support from colleagues	-1.16	0.43	-2.02– -0.30	-2.67	0.007*
Psychological support	1.27	0.56	0.16–2.37	2.26	0.02*
**Personal preventive effects**					
Prayer and spiritual activities	0.58	0.62	-0.63– 1.80	0.94	0.34
Spending time with family	0.50	0.62	-0.71– 1.73	0.81	0.41
Enough sleep	-1.04	0.63	-2.28– 0.19	-1.65	0.09
Recreation activities	-0.53	0.61	-1.74– 0.68	-0.85	0.39

## Discussion

This study provides the first systematic occupational health assessment of secondary traumatic stress among healthcare workers in a major Egyptian university hospital. The findings reveal a substantial workplace health burden with significant implications for occupational health surveillance and intervention^16^.

The results indicate that STS is quite prevalent, with 64.5% of participants above the clinical cutoff, emphasizing the significant psychological load experienced by healthcare personnel. This prevalence is consistent with the 65% global pooled prevalence observed among healthcare workers during the COVID-19 pandemic^10^ and the 70.36% prevalence found among intensive care personnel^17^. A recent study in Egypt by Hegazy et al.^6^ found stress symptoms among HCWs in newborn critical care units during the COVID-19 epidemic, but STS was not specifically assessed. Our findings add to previous work by providing precise prevalence estimates for different high-acuity departments. Elsayed et al.^18^ observed high levels of psychological distress among Egyptian health-care workers during the pandemic, with 64.8% suffering moderate to severe anxiety, which is similar with our findings.

In Saudi Arabia, Alshammari et al.^4^ found a mean STSS score of 51 (SD = 13.23) among emergency department nurses, with 27.6% reporting high levels and 50.8% reporting severe levels. Ashi et al.^19^ discovered that female healthcare workers, physicians, and those under the age of 50 were considerably more likely to develop STS in a post-pandemic group. Alonazi et al.^20^ found that 38.7% of Saudi nurses experienced moderate to severe STS. In Jordan, Al Barmawi et al.^13^ found that most critical care nurses had variable STS levels, with more than one-third suffering high to severe symptoms. Shahrour and Dardas^21^ discovered high levels of acute stress among Jordanian nurses during the epidemic. Salameh et al.^22^ discovered that 61% of emergency department nurses in Palestine had STS, reflecting the stressors seen in conflict-affected healthcare facilities.

These regional patterns indicate that STS is a continuous occupational health concern for Middle Eastern healthcare systems. However, the Egyptian context presents unique vulnerabilities, such as post-revolution healthcare system strain, ongoing economic pressures on staff retention, and limited integration of occupational mental health services into routine practice, all of which may contribute to the higher prevalence observed in the current study.

Zacharias and Upendra (2024) conducted a meta-analysis of data from 18 research including 6,844 healthcare workers and found that pooled STS prevalence ranged from 15% to 50%, depending on clinical environment and measurement methods^1^. Similarly, Thakur and Deshpande (2026) found a pooled STS prevalence of 70.36% among intensive care providers in a meta-analysis of 29 studies involving 4,925 participants^17^.

The greater percentage in our sample compared to certain global estimates could also be related to the participant makeup, which included a significant proportion of physicians (49.7%) and workers from high-acuity settings such as burn units and emergency departments. Furthermore, the post-COVID-19 pandemic scenario may have led to increased psychological burden, since healthcare systems continue to deal with cumulative trauma exposure and staffing difficulties^19^.

The detection of early warning indicators is a major opportunity for occupational health surveillance, allowing for preemptive management before STS advances to a clinically severe illness. In this study, the most endorsed warning indicators were chronic weariness, increasing fear about safety, and difficulties maintaining work-life boundaries. These symptoms are closely related to the hyperarousal and intrusion domains of STS, indicating the cumulative physiological and psychological toll of prolonged trauma exposure in the workplace^8^.

A remarkable finding is the high prevalence of these warning indicators among participants who did not match the full diagnostic criterion for STS, implying that a significant number of healthcare workers are in a subclinical state of distress.

This finding is consistent with the compassion fatigue paradigm, which proposes that STS develop gradually, beginning with early symptoms that might proceed to more severe psychological damage if not recognized and handled^14^. The strong positive correlation between STS and warning signs (*r* = 0.76) reinforces the interconnected nature of these stress responses, consistent with Ulaş and Seçer’s meta-analysis^2^, which confirmed robust correlations between STS and burnout dimensions across healthcare populations.

From the standpoint of occupational health surveillance, warning indications are useful screening indicators. They may increase accurate self-reporting during routine health examinations because they are generally less stigmatizing than official mental health diagnoses. This is especially important in the Middle East, where mental health issues are frequently seen as stigmatizing, which may deter people from getting assistance^4^. The integration of warning sign assessment into regular occupational health surveillance programs is empirically supported by these findings.

A notable finding is the strong relationship between workload parameters and STS severity. Healthcare workers who worked more than 40 h per week were more than twice as likely to develop severe STS, as were those who worked more than eight-night shifts per month. These findings are consistent with a meta-analysis by Thakur and Deshpande^17^, who identified workload and shift work as significant factors associated with compassion fatigue, and Ulaş and Seçer^2^, who confirmed strong positive correlations between occupational stressors and STS among healthcare professionals.

Physicians had considerably higher odds of STS than nurses. While both occupations are subjected to trauma, physicians may confront specific pressures such as increased decision-making responsibilities, higher expectations for therapeutic outcomes, and more exposure to moral difficulties. Ashi et al.^19^ identified physician role as a significant risk factor for STS in Saudi Arabia, while Aloufi et al.^23^ noted that healthcare social workers and physicians frequently experience higher secondary trauma levels due to the intensity of patient communications and the importance of clinical decision-making duties.

The mediation analysis found that social support played a role in mediating the association between occupational stresses and STS, accounting for 23.4% of the total effect. This finding implies that establishing social support systems inside healthcare organizations could be an effective intervention strategy. Shaqiqi and Abou El-Soud^24^ recently found that social support significantly buffered the association between STS and exhaustion among nurses caring for COVID-19 patients in the Middle East, which supports our findings. Similarly, Aloufi et al.^23^ found that perceived social support increases resilience and lowers post-traumatic stress symptoms in healthcare workers, underlining the buffering effect of collegial relationships.

Salameh et al.^22^ observed a strong correlation between social support ratings and lower STS levels among emergency nurses in Palestine. additionally, Alshammari et al.^4^ found that having a positive relationship with colleagues and receiving organizational support were strongly associated with reduced STS levels among Saudi emergency nurses, and similar themes emerged frequently in qualitative interviews. These cross-national studies consistently underscore social support as an important protective element in Middle Eastern healthcare settings.

The effectiveness of certain workplace preventative measures varies greatly. Support from coworkers, development possibilities, and financial incentives all showed strong protective relationships with lower STS levels. These findings are consistent with Zacharias and Upendra’s systematic review^1^, which identified debriefing, peer support, and organizational interventions as major protective factors against STS. Notably, psychological support services were unexpectedly associated with elevated STS score. This contradictory finding may suggest that healthcare workers in more distress are more likely to seek or use these services, rather than that the services themselves are ineffective. Only 10.7% assessed psychological support services as “very effective,” indicating that availability is limited.

This finding underlines the importance of occupational health interventions in dealing with access barriers and ensuring services are seen as confidential, relevant, and supportive particularly in Egyptian healthcare settings where mental health stigma is still prevalent. Alshammari et al.^4^ discovered in their qualitative interviews that Saudi emergency nurses frequently avoided seeking formal psychological support for fear of being perceived as “weak” or “unfit for duty,” highlighting the cultural barriers that must be overcome for such services to be effective.

Prayer and spiritual activities demonstrated a marginally significant protective role among personal preventive factors, which is in accordance with the increasing awareness of spirituality as a coping mechanism in healthcare populations. Although adequate sleep did not achieve significance in our multivariate model, Ashi et al.^19^ found appropriate sleep duration to be a significant protective factor against STS in the Saudi context. The high workload demands in our sample or variations in measurement could be the cause of this disparity.

Crucially, this multivariate model’s absence of significant protective benefits from personal preventive measures implies that, during high professional stress, individual coping mechanisms could not be enough to reduce STS. This result supports the main claim made by Alshammari et al.^4^ that organizational-level interventions are more important than individual coping strategies for addressing STS among healthcare workers. They stressed that systemic and organizational support are crucial for sustainable STS prevention, even though individual resilience is important.

### Implications for policy and practice

These results have several practical implications. First, occupational health monitoring programs for Egyptian healthcare workers especially those in high-risk departments including emergency, burn, and critical care units should incorporate routine STS screening. Finding early warning indicators is a useful way to start this type of monitoring. Second, workload management criteria, such as restrictions on working hours and night shifts, should be mandated by Egypt’s national occupational health legislation. These findings offer empirical basis for regulation reform because Egyptian healthcare professionals now routinely exceed globally approved work hour limitations. A foundation for these reforms is provided by the National Occupational Health Strategy 2024–2030^25^.

Third, preventative measures should be multimodal, integrating psychosocial support (debriefing, collegial support) with structural modifications (shift rotation, sufficient breaks). The recent finding that social support mediated about 25% of the occupational stressor-STS relationship indicates that funding collegial support structures, such peer support groups, mentorship programs, and team-building exercises, could pay off well. Fourth, Programs for occupational health must assess not just the availability of services but also their accessibility, use, and perceived efficacy by the workforce. This is especially true for psychological support services, as our investigation revealed contradictory relationships. Improving adoption and efficacy in Middle Eastern contexts requires culturally acceptable methods that guarantee confidentiality and combat stigma.

### Strengths and limitations

Strengths include a high sample size, the use of validated instruments with established internal consistency in this population, and multivariate analysis that accounts for potential confounders. The emphasis on changeable workplace characteristics makes the findings useful for occupational health policy.

Some limitations should be pointed out. The cross-sectional design limits out causal inferences about the direction of correlations between occupational characteristics and STS. Longitudinal studies are required to establish temporal ordering and assess preventative intervention efficacy over time. The sample, while large, was chosen from a single healthcare system, which may limit its applicability to other contexts with differing organizational cultures and resources. Furthermore, relying on convenience sampling may introduce selection bias, since those who are distressed are more likely to participate, thus altering prevalence estimates. Furthermore, relying on self-report measures increases the likelihood of common method bias and social desirability effects.

## Conclusions

This study indicates that STS is frequent among Egyptian healthcare workers and is closely linked to modifiable occupational factors such as long working hours, frequent night shifts, and the physician role. Social support partially mediates the association between occupational stresses and STS, emphasizing the value of collegial ties in reducing workplace stress. Collegial support, development options, financial incentives, and shift rotation are among occupational preventive strategies related to decreased STS. These findings are consistent with and extend a growing body of literature from Middle Eastern countries that identify workload, professional role, and social support as significant determinants in STS development. As the first systematic occupational health assessment of STS in Egypt, this study establishes a critical baseline for benchmarking and informs evidence-based occupational health policy development, while highlighting the urgent need for culturally sensitive, multi-level interventions to address the psychological well-being of healthcare workers in the region.

## Data Availability

All data supporting the findings of this study are available from the corresponding author upon reasonable request.

## Abbreviations

AOR: Adjusted odds ratio
CI: Confidence interval
COVID-19: Coronavirus disease 2019
HCWs: Healthcare workers
I-CVI: Item-level content validity index
JD-R: Job demands-resources model
PTSD: Post-traumatic stress disorder
SPSS: Statistical Package for Social Sciences
STS: Secondary traumatic stress
STSS: Secondary Traumatic Stress Scale
